# Multimorbidity and the use of health services in the Brazilian population: National Health Survey 2019

**DOI:** 10.1590/S2237-96222023000300007.en

**Published:** 2023-10-30

**Authors:** Ana Sara Semeão de Souza

**Affiliations:** 1 Universidade Estácio de Sá, Faculdade de Medicina, Rio de Janeiro, RJ, Brazil

**Keywords:** Health Services, Multimorbidity, Cross-Sectional Studies, Epidemiological Surveys, Chronic disease, Servicios de Salud, Multimorbilidad, Estudios Transversales, Encuestas Epidemiológicas, Enfermedad crónica, Serviços de Saúde, Multimorbidade, Estudos Transversais, Inquéritos Epidemiológicos, Doença Crônica

## Abstract

**Objective:**

To describe the prevalence of health service use due to multimorbidity according to sociodemographic and health characteristics of the Brazilian population; to analyze the relationship between multimorbidity and the use of health services.

**Methods:**

This was a cross-sectional study using data from the 2019 National Health Survey. The outcomes were seeking health services in the last 15 days, medical consultation and hospitalization in the previous 12 months. Multimorbidity was defined as ≥ 2 chronic diseases. Associations were assessed using Poisson regression.

**Results:**

Of the 81,768 individuals, prevalence of seeking health services among individuals with multimorbidity was 38.0% higher (95%CI 1.31;1.45), medical appointments, 11.0% higher (95%CI 1.10;1.12), and 56.0% higher for hospitalizations (95%CI 1.44;1.70), compared to those without multimorbidity. This relationship was higher for seeking health services and medical appointments among male.

**Conclusion:**

The use of health services was higher among those with multimorbidity, but different between the types of health services used and sexes.

## Study contributions

**Main results:**

Having multimorbidity increased the use of health services, even after progressive adjustment by sociodemographic characteristics and health needs. This relationship was greater among males for medical consultations.

**Implications for services:**

Greater use of health services by individuals with multiple non-communicable diseases (NCDs) points to the need for changes in care models, with focus on continuity of care.

**Perspectives:**

Health services should focus on continuous, coordinated and comprehensive approaches to the care of people with multimorbidity, thus seeking to increase the efficiency and quality of care provided to this population.

## RESUMEN

**Objetivo:**

Describir prevalencia de uso de servicios de salud por multimorbilidad según características sociodemográficas de salud de población brasileña; analizar relación entre multimorbilidad y utilización de servicios de salud.

**Métodos:**

Estudio transversal con datos de Encuesta Nacional de Salud de 2019.

**Resultados:**

fueron búsqueda de servicios de salud en últimos quince días; consulta médica y hospitalizaciones en últimos doces meses. Multimorbilidad se definió como ≥ 2 enfermedades crónicas. Asociaciones se evaluaron mediante regresión de Poisson.

**Resultados:**

De los 81.768 individuos, prevalencia de búsqueda de servicios de salud entre los individuos con multimorbilidad fue 38,0% mayor (IC_95_% 1,31;1,45), citas médicas 11,0% mayor (IC_95_% 1,10;1,12) y 56,0% mayor para hospitalizaciones (IC_95_% 1,44;1,70), en comparación con aquellos sin multimorbilidad. Esta relación fue mayor para búsqueda y citas médicas entre el sexo masculino.

**Conclusión:**

El uso de los servicios de salud fue mayor entre aquellos con multimorbilidad, pero diferente entre los tipos de servicios de salud utilizados y sexos.

## RESUMO

**Objetivo:**

Descrever a prevalência do uso de serviços de saúde por multimorbidade segundo características sociodemográficas e saúde da população brasileira, e analisar a relação entre a multimorbidade e o uso de serviços de saúde.

**Métodos:**

Estudo transversal utilizando dados da Pesquisa Nacional de Saúde 2019. Os desfechos foram busca por serviços de saúde nos últimos 15 dias, consulta médica e internações nos últimos 12 meses. Multimorbidade foi definida como ≥ 2 doenças crônicas. As associações foram avaliadas pela regressão de Poisson.

**Resultados:**

Dos 81.768 indivíduos, a prevalência de busca por serviços de saúde entre indivíduos com multimorbidade foi 38% maior (IC_95_% 1,31;1,45), consultas médicas, 11% maior (IC_95_% 1,10;1,12), e 56% maior para internações (IC_95_% 1,44;1,70), em comparação àqueles sem multimorbidade. Essa relação foi maior para busca e consultas médicas no sexo masculino.

**Conclusão:**

O uso de serviços de saúde foi maior entre aqueles com multimorbidade, mas diferente entre os tipos de serviços de saúde utilizados e sexos.

## INTRODUCTION

Multimorbidity is often defined as the co-occurrence of multiple chronic medical conditions in the same person.^1-3^ The progressive increase in individuals with multimorbidity is recognized as one of the main challenges for health professionals and public health systems.^4,5^

In Brazil, prevalence of multimorbidity was estimated at 24.2% in 2013, being more common in women, among those with less education and older individuals.^6^ Furthermore, it was found that in Brazil people develop multimorbidity at a younger age than people in wealthier countries, and women, ten years earlier than men.^6^

Souza & Braga (2020) found that in Brazil in 2013, prevalence of people with multimorbidity seeking health services was two times greater in relation to those without multimorbidity.^7^ That study also found that presence of multimorbidity increased the prevalence of medical consultations by 16% among men and 11% among women; and hospitalizations by 55% among men and 45% among women in 2013.^7^ In view of high demand for health services, case management programs for individuals with multimorbidity are being developed and evaluated around the world, but there are still no Brazilian guidelines on what constitutes the best care for these people.^8^

Therefore, knowing the main determinants of multimorbidity and its association with different health services contributes to the development of effective health protocols and policies, which aim at continuous, coordinated and comprehensive, person-centered approaches.^9^ Despite high prevalence of multimorbidity in Brazil, few published studies have examined its relationship with health services for the entire Brazilian population.^7,10,11^

As such, this study sought to describe the prevalence of the use of different health services due to multimorbidity according to sociodemographic and health characteristics of the Brazilian population, as well as to analyze association between them. 

## METHODS

This was a cross-sectional study using data from the National Health Survey (*Pesquisa Nacional de Saúde* - PNS) conducted in 2019. The PNS survey was carried out by the Brazilian Institute of Geography and Statistics (*Instituto Brasileiro de Geografia e Estatística* - IBGE) in partnership with the Ministry of Health. The survey sampling was carried out in three selection stages and is representative of the Brazilian population. Details of the sampling process can be accessed in a specific publication.^12^ For the purposes of this article, the study population was comprised of people aged 18 or over.

Health service use was analyzed through three outcomes: seeking health services in the last 15 days (excluding dental services), medical consultation and hospitalization, both in the last 12 months. The seeking health services and hospitalization outcomes were assessed dichotomously (yes; no), while medical consultations in the last 12 months, were assessed through the following question: *When did you last consult a doctor*? With the answer option being (i) up to one year ago, (ii) more than one year and less than two years ago, (iii) more than two years and less than three years ago, (iv) more than three years ago and (v) never consulted a doctor. Affirmative answers to item (i) were classified as “yes” and options (ii, iii, iv and v) were classified as “no”.

Exposure was assessed by asking the question: *Has a doctor ever diagnosed you as having ...*? Except for chronic back problems, which were assessed by asking the question: *Do you have a chronic back problem, such as chronic back or neck pain, low back pain, sciatica, vertebrae or disc* *problems?* ^13^ The number of morbidities was counted based on the following morbidities: chronic back problem (chronic back or neck pain, low back pain, sciatica, vertebrae or disc problems), arthritis or rheumatism, cancer, diabetes, asthma or asthmatic bronchitis, hypertension (excluding hypertension when only during pregnancy), heart disease (heart attack, angina, heart failure or other), chronic kidney failure, depression and work-related musculoskeletal disorders (WMSDs). All self-reported morbidities had a weight of 1 in the morbidity count. Multimorbidity was defined by the presence of two or more self-reported morbidities.

The sociodemographic characteristics analyzed were: sex (female; male), age categorized in years (18-29; 30-39; 40-49; 50-59; 60-69; ≥ 70), education (no education; incomplete elementary education; complete elementary education; incomplete high school education; complete high school education; incomplete higher education; complete higher education) and self-reported race/skin color (White; mixed race; Black; Asian; Indigenous). 

The variables related to health services were: having health insurance (yes; no) and being registered with Family Health Strategy (FHS) teams (yes; no). Health needs were assessed through self-rated state of health (very good; good; fair; poor; very poor) and limitation of habitual activities in the last 15 days (yes; no).

Prevalence rates and 95% confidence intervals (95%CI) were estimated for each health service use outcome, stratified by multimorbidity and according to the independent variables.

Crude and adjusted Poisson regression models were used to estimate the measures of effect, prevalence ratios (PR) and their respective 95%CI. The models were adjusted for the independent variables for each health service use and multimorbidity outcome. In the general population, the use of health services is strongly motivated by the person’s sex.^15,16^ As such, the analyses were stratified by sex.

Surveys that use complex sampling have different probabilities of selecting individuals and clusters. The survey module of the Stata SE software version 15.0 was used to perform the analyses, taking into account the sampling parameters and weights of the individuals. 

The PNS was approved by the National Research Ethics Commission (Process No. 3.529.376, dated August 23, 2019). A consent form was signed by all participants at the time of the interview. The data relating to the PNS are publicly accessible, made available electronically by the IBGE, and preserve the identity of the survey participants. The data were extracted and analyzed in July 2022. 

## RESULTS

Of the 81,768 individuals included in the study, 53.2% were female, 22.8% were between 18 and 29 years of age, the majority were Black and of mixed race (54.3%), and 29.6% reported having completed high school. Of the population assessed, 27% had health insurance and 61.5% were registered with a Family Health Strategy team. Regarding the health of the population, 52.7% self-rated their health as good and 8.5% reported that their habitual activities had been limited in the last 15 days.

Prevalence of multimorbidity was 24.1% (95%CI 23.6;24.7). We found that 22.1% (95%CI 21.6;22.7) reported having sought some sort of health service in the last 15 days, 80.7% (95%CI 80.2;81.1) had had a medical consultation in the last 12 months and 7.6% (95%CI 7.2;8.0) had been hospitalized in the last 12 months. 

 Prevalence of seeking health services in the last 15 days was 37.8% (95%CI 36.6;39.0) among individuals with multimorbidity and 40.1% among females (95%CI 38.7;41.5). Among individuals with multimorbidity, prevalence of seeking health services was higher among those with incomplete higher education (44.8%; 95%CI 37.6;52.2) and complete higher education (42.9; 95%CI 39.6;46.3), compared to those with less education. Higher prevalence of seeking health services was found among individuals with multimorbidity, those who had health insurance (42.4%; 95%CI 40.0;44.8), those who self-rated their state of health as very poor (53.6%; 95%CI 47.6;59.5) and among those who reported some limitation of habitual activities in the last 15 days (65.7%; 95%CI 63.4;68.0) (Table 1).

**Table 1:** Prevalence of seeking health services in the last 15 days, medical consultations and hospitalizations in the last 12 months, stratified by multimorbidity, according to sociodemographic and health characteristics, National Health Survey, Brazil, 2019

Variables	Sought health services^c^		Medical consultations in the last 12 months^c^		Hospitalizations in the last 12 months^c^	
	**Without multimorbidity**	**Multimorbidity**	**Without multimorbidity**	**Multimorbidity**	**Without multimorbidity**	**Multimorbidity**
	**% (95%CI)^a^**	**% (95%CI)^a^**	**% (95%CI)^a^**	**% (95%CI)^a^**	**% (95%CI)^a^**	**% (95%CI)^a^**
Overall	18.3 (17.8;18.9)	37.8 (36.6;39.0)	78.8 (78.3;79.4)	94.9 (94.4;95.4)	6.0 (5.7;6.4)	14.0 (13.1;14.9)
**Sex**						
Male	15.0 (14.3;15.8)	34.0 (32.1;36.0)	73.0 (72.0;73.9)	93.2 (92.2;94.1)	4.9 (4.4;5.4)	14.0 (12.7;15.3)
Female	21.5 (20.7;22.4)	39.7 (38.3;41.2)	84.5 (83.7;85.2)	95.8 (95.1;96.3)	7.2 (6.7;7.7)	14.0 (12.8;15.3)
**Age**						
18-29	15.8 (15.6;17.1)	39.0 (32.7;45.7)	76.5 (75.2;77.8)	90.5 (86.4;93.4)	6.1 (5.4;6.8)	16.1 (11.6;21.8)
30-39	17.3 (16.2;18.4)	37.5 (33.4;41.7)	77.5 (76.4;78.6)	92.1 (89.3;94.2)	6.4 (5.8;7.1)	16.7 (12.9;21.4)
40-49	18.8 (17.6;20.0)	40.8 (37.7;44.0)	78.5 (77.2;79.7)	94.9 (93.5;96.0)	5.3 (4.7;6.0)	12.2 (10.4;14.4)
50-59	19.3 (17.9;20.7)	37.0 (34.5;39.5)	79.8 (78.5;81.0)	94.0 (92.8;95.0)	5.7 (4.5;7.4)	12.4 (11.0;14.0)
60-69	21.8 (20.1;23.5)	38.0 (35.8;40.3)	82.4 (81.0;83.8)	96.5 (95.7;97.2)	5.9 (5.0;7.0)	13.4 (12.0;15.0)
= 70	24.2 (22.2;26.3)	36.2 (34.1;38.5)	87.2 (85.7;88.5)	96.1 (95.3;96.8)	7.6 (6.5;8.9)	16.3 (14.7;18.0)
**Race/skin color**						
White	19.2 (18.7;20.2)	38.5 (36.6;40.4)	81.3 (80.5;82.2)	95.5 (94.7;96.2)	5.8 (5.3;6.3)	13.8 (12.6;15.2)
Black	18.9 (17.2;20.7)	38.2 (34.8;41.7)	78.1 (76.3;79.9)	94.8 (93.4;96.0)	5.4 (4.6;6.3)	13.7 (11.5;16.4)
Asian	24.3 (16.6;34.1)	38.6 (25.3;53.8)	80.6 (74.8;85.3)	99.4 (97.5;99.8)	4.5 (2.6;7.6)	16.4 (7.3;32.7)
Mixed race	17.2 (16.5;18.0)	36.9 (35.2;38.7)	76.6 (75.7;77.5)	94.0 (93.2;94.8)	6.5 (5.9;7.2)	14.3 (13.1;15.6)
Indigenous	15.8 (10.6;23.0)	34.3 (23.4;47.2)	70.1 (54.9;81.8)	93.9 (86.4;97.4)	4.9 (2.6;9.1)	10.0 (4.5;20.5)
**Level of education**						
No education	16.5 (14.7;18.4)	36.5 (33.3;40.0)	76.9 (74.5;79.0)	96.0 (95.0;96.9)	6.5 (5.2;8.0)	19.7 (17.1;22.6)
Incomplete elementary education	18.3 (17.3;19.4)	36.7 (34.8;38.5)	76.1 (74.9;77.2)	94.9 (94.1;95.7)	6.2 (5.3;7.2)	14.0 (12.6;15.2)
Complete elementary education	16.6 (14.7;18.7)	38.9 (34.7;43.3)	75.0 (72.8;77.0)	94.5 (92.3;96.1)	6.2 (5.0;7.6)	12.7 (10.4;15.4)
Incomplete high school education	17.2 (15.0;19.5)	36.2 (30.5;42.4)	73.2 (70.7;75.6)	93.3 (89.4;95.8)	6.5 (5.1;8.2)	11.1 (8.1;15.1)
Complete high school education	17.1 (16.1;18.2)	36.4 (33.6;39.3)	79.1 (78.0;80.1)	94.5 (93.2;95.6)	6.0 (5.4;6.6)	13.0 (11.1;15.3)
Incomplete higher education	19.5 (17.1;22.0)	44.8 (37.6;52.2)	79.6 (77.1;81.9)	92.1 (87.0;95.4)	5.0 (4.0;6.1)	13.0 (9.2;18.1)
Complete higher education	21.9 (20.5;23.3)	42.9 (39.6;46.3)	86.3 (85.2;87.3)	95.7 (94.4;96.8)	6.0 (5.3;6.9)	13.7 (11.7;15.9)
**Health Insurance**						
Yes	24.0 (22.7;25.3)	42.4 (40.0;44.8)	90.2 (89.4;90.9)	97.5 (96.7;98.0)	7.8 (7.1;8.6)	14.6 (13.0;16.3)
No	16.2 (15.5;16.8)	35.8 (34.5;37.2)	74.4 (73.7;75.2)	93.8 (93.1;94.4)	5.4 (5.0;5.8)	13.8 (12.8;14.7)
**Registered with FHS^b^**						
Yes	18.4 (17.7;19.2)	38.6 (37.1;40.0)	78.5 (77.8;79.3)	94.9 (94.2;95.5)	6.1 (5.7;6.5)	14.5 (13.5;15.6)
No	18.7 (17.6;20.0)	37.3 (34.7;40.0)	79.9 (78.8;81.1)	95.2 (94.1;96.1)	6.4 (5.5;7.3)	12.8 (11.3;14.6)
**Self-rated state of health**						
Very good	14.4 (13.2;15.7)	25.2 (21.0;30.0)	76.2 (74.7;77.7)	93.3 (90.4;95.4)	4.6 (3.9;5.3)	7.5 (5.0;11.0)
Good	15.9 (15.2;16.7)	32.7 (30.6;34.9)	77.6 (76.8;78.3)	93.8 (92.8;94.8)	5.1 (4.7;5.5)	10.2 (8.8;11.9)
Fair	25.8 (24.5;27.1)	39.2 (37.5;40.8)	83.1 (82.1;84.2)	95.3 (94.6;95.9)	8.9 (7.8;10.1)	13.8 (12.7;15.0)
Poor	36.4 (32.6;40.3)	48.5 (45.2;51.8)	89.6 (87.1;91.6)	96.7 (95.5;97.6)	13.0 (10.7;15.7)	23.8 (20.7;27.2)
Very poor	35.9 (29.0;43.3)	53.6 (47.6;59.5)	88.8 (82.2;93.1)	95.9 (91.2;98.1)	21.4 (15.9;28.0)	30.3 (25.3;35.9)
**Limitation of habitual activities in the last 15 days**						
Yes	62.6 (60.1;65.0)	65.7 (63.4;68.0)	93.6 (92.4;94.5)	97.7 (96.7;98.3)	18.2 (16.0;20.5)	25.4 (23.3;27.6)
No	15.5 (15.0;16.0)	31.2 (29.9;32.6)	77.9 (77.3;78.5)	94.2 (93.6;94.8)	5.3 (4.9;5.6)	11.3 (10.5;12.2)

a) 95%CI: 95% confidence interval; b) FHS: Family Health Strategy; c) Percentage takes sampling weights into account: *svy*.

Among people with multimorbidity, prevalence of consultations in the last year was 94.9%, while it was 78.8% for individuals without multimorbidity. Among individuals with multimorbidity, prevalence of having medical consultations in the last year was higher among females (95.8%; 95%CI 95.1;96.3), among people aged 60 to 69 years (96.5%; 95%CI 95.7;97.2), those with health insurance (97.5%; 95%CI 96.7;98.0), those who self-rated their state of health as very poor (95.9%; 95%CI 91.2;98.1) and those whose habitual activities had been limited (97.7%; 95%CI 96.7;98.3) (Table 1).

Prevalence of hospitalizations in the last year among individuals with multimorbidity was 14%, while it was 6% among those without multimorbidity. Hospitalizations in the last year among individuals with multimorbidity were more frequent among people aged 30 to 39 years (16.7%; 95%CI 12.9;21.4) and who had no education (19.7%; 95%CI 17.1;22.6). Prevalence of hospitalization among people with multimorbidity showed an inverse relationship with self-rated state of health, being 7.5% (95%CI 5.0;11.0) among those with very good self-rated health and 30.3% (95%CI 25.3;35.9) for those with very poor self-rated health (Table 1).

Table 2 shows the crude and adjusted analysis of association between multimorbidity and use of health services (seeking health services, medical consultations and hospitalizations) for the general population and stratified by sex. The adjusted model showed significant association between multimorbidity and use of services. Prevalence of seeking health services was 38% higher among people with multimorbidity compared to those who did not have multimorbidity, in the general population (PR = 1.38; 95%CI 1.31;1.45). Regarding the sex of the participants, seeking health services, among those with multimorbidity, was 46% for males (PR = 1.46; 95%CI 1.34;1.60) and 38% for females (PR = 1.38; 95%CI 1.30;1.46).

**Table 2:** Crude and adjusted analysis comparing multimorbidity and use of general health services, stratified by sex, National Health Survey, Brazil, 2019

Variables	Geral		Males		Females	
	**Crude PR^a^** **(95%CI)^c^**	**Adjusted PR^b^** **(95%CI)^c^**	**Crude PR^a^** **(95%CI)^c^**	**Adjusted PR^b^** **(95%CI)^c^**	**Crude PR^a^** **(95%CI)^c^**	**Adjusted PR^b^** **(95%CI)^c^**
Sought health services	2.06	1.40	2.26	1.46	1.85	1.38
	(1.97;2.15)	(1.33;1.48)	(2.10;2.44)	(1.34;1.60)	(1.75;1.95)	(1.30;1.46)
Consultations in the last 12 months	1.20	1.11	1.28	1.16	1.13	1.09
	(1.19;1.21)	(1.10;1.12)	(1.26;1.30)	(1.14;1.18)	(1.12;1.14)	(1.07;1.10)
Hospitalizations in the last 12 months	2.32	1.59	2.86	1.47	1.96	1.74
	(2.14;2.51)	(1.44;1.75)	(2.46;3.33)	(1.22;1.79)	(1.75;2.20)	(1.52;2.00)

a) Crude PR: Crude prevalence ratio, taking the study sampling weights into account: *svy*; b) Adjusted PR: Prevalence ratio adjusted for age, level of education and race/skin color, having health insurance, being registered with the family health strategy, self-rated state of health and limitation of habitual activities, taking the study sampling weights into account: *svy*; c) 95%CI: 95% confidence interval.

Among individuals with multimorbidity, prevalence of medical consultations in the last 12 months was 11% higher (95%CI 1.10;1.12), compared to those without multimorbidity. In the case of males, prevalence of medical consultations was higher among individuals with multimorbidity (PR = 1.17; 95%CI 1.15;1.18) than for females (PR = 1.09; 95%CI 1.07;1.10), compared to those who did not have multimorbidity. Finally, prevalence of hospitalizations among individuals with multimorbidity was 56% higher (95%CI 1.44;1.70), compared to people without multimorbidity. The prevalence of hospitalizations in the last 12 months, for those with multimorbidities, were 74% higher among females (PR = 1.74; 95%CI 1.52;2.00) and 47% higher among males (PR = 1.47 ; 95%CI 1.22;1.79) (Table 2).

## DISCUSSION

This article identified association of multimorbidity with use of health services, being greater among males for medical consultations. Among individuals with multimorbidity, seeking health services in the last 15 days and hospitalizations in the last 12 months were twice as high, compared to individuals without multimorbidity. Prevalence of having medical consultations in the last 12 months was 20% higher among those with multimorbidity. 

The study conducted by Shi et. al. (2021)^11^ found that the demand for health services and hospitalizations was greater among individuals with multimorbidity between 1998 and 2013, in Brazil. The results of our study point to continued greater use of a variety of health services in Brazil, in 2019, among people with multimorbidity, emphasizing the need for expanded discussions on the provision of services for this population.

A study using data from the 2013 PNS found that prevalence of multimorbidity was 22.2%, based on a list of diseases similar to that used in our study. In the former study, 16.6% of the population had sought health services in the 15 days prior to the survey, 71.8% had had medical consultations and 6.6% had been hospitalized in the last 12 months.^7^ The findings for 2019 show a slight increase in the use of healthcare services in recent years. 

A recent systematic review estimated combined global prevalence of multimorbidity to be 33.1%. That review included 70 studies, which had analyzed 37 high-income countries and 35 low- and middle-income countries, from 1992 to 2017. In high-income countries estimated prevalence of multimorbidity was 37.9% , while in low- and middle-income countries it was 29.7%.^17^ However, it is still unclear whether there is geographic variation in the prevalence of multimorbidity or whether these differences can be simply explained by differences in diagnostic and data management systems between countries with different income levels.^17^

In our study, among individuals with multimorbidity, seeking health services was higher among younger people. Although multimorbidity has higher prevalence in older age groups, in Brazil people develop morbidities and multimorbidity at a younger age than people in wealthier countries.^5,6,18^ Being female and being an older adult are the factors that most bear influence on the population using health services.^19,20^

Prevalence of consultations was higher among people with multimorbidity compared to individuals without multimorbidity. However, among individuals with multimorbidity, prevalence of health service use does not differ much between sociodemographic characteristics, access and health needs. In the general population, health services are more used by women, people with a higher level of education and those with health insurance.^16^ However, a study conducted in Serbia,^21^ in 2013, found that having multimorbidity reduced differences in the prevalence of medical consultations with regard to these variables, indicating a possible reduction in differences in the use of health services among people with health needs, such as people with multimorbidity.

Some studies indicate that, although the underlying reasons for association found between education, deprivation and multimorbidity are probably complex and multifactorial, intermediary factors such as lifestyle, access to and use of health services and neighborhood context are also important.^22,23^ Social inequities are revealed through the positive relationship between multimorbidity and poorer socioeconomic indicators, pointing to the challenge to be faced by health systems in promoting this population’s access to health services.^5^

Even after progressive adjustment of sociodemographic characteristics, access and health needs, having multimorbidity increased the use of health services, with greater influence for the male sex for medical consultations. These findings corroborate the results of studies previously carried out in Brazil (2020),^7^ China (2014),^23^ Ireland (2011),^24^ and the Netherlands (2014),^25^ demonstrating an increase in primary and secondary care use associated with multimorbidity, even when controlling for age, sex and social status.

The limitations of this study include the use of self-reported measures of morbidity and health services that may underestimate their prevalence.^2,18^ However, in population-based studies, self-reported information is considered valid and robust for monitoring the health situation.^26,27^ Furthermore, this study considered all diseases equally, although the effect of multimorbidity on individuals may vary depending on chronic noncommunicable disease (NCDs) combination and severity.^5^ It is important to highlight that our multimorbidity classification only used the set of ten health conditions, which may have led to it being underestimated among the study population. Variability in the number of health conditions included in multimorbidity classification measures makes it difficult to compare prevalence and its impact across populations.^2,28^ Another important limitation related to the cross-sectional study design is reverse causality. Temporality between medical diagnosis and seeking health services cannot be defined, therefore, the results must be interpreted paying attention to the possible overestimation of the measures of effect between exposure and outcome, given that for presence/absence of morbidity to be self-reported, diagnosis by a health professional is necessary, implying the use of health services.

Among its strengths, this study used nationwide data, enabling the generalization of its results to the entire Brazilian population and even to countries with equivalent characteristics. Furthermore, periodic surveys may provide important information for monitoring the use of health services among this population.

In conclusion, this study showed that people with multimorbidity used health services more, regardless of the type of service, considering that they can often be attended to by several health professionals, given the cross-cutting format of service provision and health policies that currently exist for the care of people with NCDs. In this sense, health care policies and care provision actions focused on access to health services must be developed for this population, breaking the traditional and cross-cutting approaches to specialist care, transforming them into actions focused on the health service user and not on their individual diseases. 

Care for chronic conditions requires much more complex care models, in which the focus is not on response time depending on risks, but on continuity of care without its being fragmented.^29^ In this sense, prospective studies can help to deepen knowledge about the causal relationship between multimorbidity patterns and their relationship with health services, as well as the creation of more efficient care models, thus seeking to increase the quality of care provided to this population. 
